# The long wave of COVID-19: a case report using Imagery Rehearsal Therapy for COVID-19-related nightmares after admission to intensive care unit

**DOI:** 10.3389/fpsyg.2023.1144087

**Published:** 2023-05-18

**Authors:** Giada Rapelli, Giorgia Varallo, Serena Scarpelli, Giada Pietrabissa, Alessandro Musetti, Giuseppe Plazzi, Christian Franceschini, Gianluca Castelnuovo

**Affiliations:** ^1^Department of Medicine and Surgery, University of Parma, Parma, Emilia-Romagna, Italy; ^2^Department of Psychology, Sapienza—University of Rome, Rome, Italy; ^3^Psychology Research Laboratory, Istituto Auxologico Italiano IRCCS, Milan, Lombardy, Italy; ^4^Faculty of Psychology, Catholic University of the Sacred Heart, Milan, Milan, Lombardy, Italy; ^5^Department of Humanities, Social Sciences and Cultural Industries, University of Parma, Parma, Emilia-Romagna, Italy; ^6^IRCCS Institute of Neurological Sciences of Bologna (ISNB), Bologna, Emilia-Romagna, Italy; ^7^Department of Biomedical, Metabolic and Neural Sciences, University of Modena and Reggio Emilia, Modena, Emilia-Romagna, Italy

**Keywords:** case report, imagery rehearsal therapy, intensive care unit, COVID-19, SARS-CoV-2, nightmare, sleep disorder

## Abstract

**Introduction:**

The COVID-19 pandemic caused several psychological consequences for the general population. In particular, long-term and persistent psychopathological detriments were observed in those who were infected by acute forms of the virus and need specialistic care in the Intensive Care Unit (ICU). Imagery rehearsal therapy (IRT) has shown promising results in managing nightmares of patients with different traumas, but it has never been used with patients admitted to ICUs for severe COVID-19 despite this experience being considered traumatic in the literature.

**Methods:**

The purpose of this case study is to describe the application of a four-session IRT for the treatment of COVID-related nightmares in a female patient after admission to the ICU. A 42-year-old Caucasian woman who recovered from a pulmonary rehabilitation program reported shortness of breath, dyspnea, and everyday life difficulties triggered by the long-COVID syndrome. She showed COVID-related nightmares and signs of post-traumatic symptoms (i.e., hyperarousal, nightmares, and avoidance of triggers associated with the traumatic situation). Psychological changes in the aftermath of a trauma, presence, and intensity of daytime sleepiness, dream activity, sleep disturbances, aspects of sleep and dreams, and symptoms of common mental health status are assessed as outcomes at the baseline (during the admission to pneumology rehabilitation) at 1-month (T1) and 3-month follow-up (T2). Follow-up data were collected through an online survey.

**Results:**

By using IRT principles and techniques, the patient reported a decrease in the intensity and frequency of bad nightmares, an increase in the quality of sleep, and post-traumatic growth, developing a positive post-discharge.

**Conclusion:**

Imagery rehearsal therapy may be effective for COVID-19-related nightmares and in increasing the quality of sleep among patients admitted to the ICU for the treatment of COVID-19. Furthermore, IRT could be useful for its brevity in hospital settings.

## Introduction

The COVID-19 pandemic and confinement measures are established adverse events increasing the risk of anxiety, fear of contagion, depression, and post-traumatic stress disorder (PTSD; Giusti et al., 2020, 2022; Rapelli et al., 2020; Rossi et al., 2020; Pietrabissa et al., 2021). Changes in daily routine and work schedule also caused alterations in the sleep–wake cycle and decreased sleep quality (Franceschini et al., 2020). Insomnia, hypersomnia, and nightmares were frequently observed during the COVID-19 pandemic (Borghi et al., 2021; Margherita et al., 2021; Scarpelli et al., 2022a) and, especially during the second wave, people presenting with more nightmares also showed greater sleep problems and higher levels of PTSD (Scarpelli et al., 2021).

Individuals suffering from COVID-19 post-acute symptoms reported greater sleep alterations and more frequent nightmares than individuals with “short-COVID” (Scarpelli et al., 2022b). Furthermore, patients admitted to the intensive care unit (ICU) (e.g., patients intubated and mechanically ventilated due to respiratory failure) were at higher risk of experiencing PTSD symptoms including intrusion, avoidance, negative alterations in cognition and mood, arousal, and reactivity (Nagarajan et al., 2022); in fact, ICU represented additional extreme stressors for patients with COVID-19, who could feel fear of death, pain from medical procedures such as endotracheal intubation, limited ability to communicate, feelings of loss of control, mood swings, sleep disturbance, and feelings of panic and suffocation (Kaseda and Levine, 2020).

Acute phase survivors of severe COVID-19 treated in the ICU were more likely to experience poor sleep quality characterized by wakefulness, a high proportion of time spent in shallow sleep, and a relatively low proportion of time spent in REM sleep, as well as vivid nightmares and hallucinations (Helms et al., 2020; Rogers et al., 2020). This could impact their recovery process in the long-term (e.g., post-intensive care syndrome and long-COVID syndrome) from 20 days after the ICU discharge (Weidman et al., 2022) to 3-month follow-up (Rousseau et al., 2021) and persist even after 1 year from discharge (Kaseda and Levine, 2020).

One year after admission to an ICU, patients tend to develop a worse health-related quality of life, persistent dyspnea, and impairment in pulmonary function (Gamberini et al., 2021). ICU might, therefore, be considered a potential high-trauma experience for patients with COVID-19 (Beck et al., 2021; Janiri et al., 2021), and its consequences deserve appropriate attention. In this respect, imagery rehearsal therapy (IRT; Kellner et al., 1992), a third-generation cognitive-behavioral technique, has shown promising results in reducing the number and intensity of nightmares in patients who experienced different types of traumas (Krakow and Zadra, 2006; Pierpaoli-Parker et al., 2021; Poschmann et al., 2021) and only partially in ICU patients (Szabó and Tóth, 2021). It works by elaborating on the original nightmare and providing a cognitive shift that empirically refutes the original premise of the nightmare (Lancee et al., 2011; Germain, 2013). To date, to the best of our knowledge, no studies have investigated the efficacy of IRT in the treatment of trauma-related experiences of COVID-19.

The purpose of this case study is to describe the implementation of four-session IRT for the treatment of a female patient previously admitted for a 3-week ICU treatment following COVID-19 and showing trauma-related nightmares 9 months after admission. Furthermore, this case study intends to illustrate for the first time how IRT’s skills and strategies can be used for the treatment of post-traumatic nightmares after admission to an ICU for COVID-19.

CARE guidelines (Case Report; Gagnier et al., 2013) for writing a patient case report in a checklist were used to enhance the manuscript process (see Supplementary data sheet 2).

## Patient information

Grace (pseudonym) was admitted to the 1-month of a pulmonary rehabilitation program with complaints of shortness of breath, dyspnea, and everyday life difficulties triggered by long-COVID syndrome (she received the diagnosis of COVID-19 9 months earlier), which also caused her significant weight gain due to sedentariness (current body mass index, BMI = 29.8 kg/m^2^). She was a 42-year-old woman living in Central Italy. She worked as an employee, was married, and had two children (a 12-year-old girl and a 7-year-old boy). During the initial assessment phase, no particular stressful family, couple, or work situations emerged. During the present hospitalization, Grace received a diagnosis of obstructive sleep apnea syndrome (OSAS) and received continuous positive airway pressure (CPAP) therapy with benefits. Grace reported using CPAP regularly during the initial adjustment in the hospital. Still, she continued to experience sleep disturbances and daily nightmares once a day (intrusive thoughts/memories pertained to the doctors’ communication that she would be intubated in ICU because her condition worsened after contracting the virus).

For this reason, the patient asked for psychological support. No pharmacological treatment was used. Ethical approval of the study was obtained by the Medical Ethics Committee of the IRCCS Istituto Auxologico Italiano (ID: 2021_03_23_02). All procedures performed in the study were run following the ethical standards of the institutional and/or national research committee and with the Helsinki Declaration and its later amendments or comparable ethical standards.

Before the study, the interviewer informed the participant about the aim and procedure of the interview and intervention and obtained her signed informed consent and permission to audio-record the session for research purposes (see Supplementary data sheet 1).

It is to be noted as an unanticipated event that, at the final debriefing with the psychologist following the last survey (T2), Grace reported a stressful situation in the family that led to her decision to separate from her husband. The husband and the wife in the last survey were separated at home.

## Diagnostic assessment

### Qualitative measure: semi-structured interview

A trained psychologist (GR) conducted a face-to-face semi-structured interview with the patient during the first week of pulmonary rehabilitation in order to explore the deep experience of Grace in the ICU.

The semi-structured interview occurred in a dedicated room of the hospital, lasted for about 1 h, and, during the interview, Grace was asked about the experience of being infected and the experience of the ICU, feelings and worries about COVID-19 management during the hospitalization and at home, feelings about COVID-19 consequences during hospitalization and at home after discharge, and the frequent nightmares related to the experience in ICU.

A storytelling approach with probing questions (e.g., “tell me more about that experience” and “how did that make you feel?”) was further used during the interview to clarify or expand meanings presented by the patient, thus facilitating a dialogic interaction process and to help to express deepest thoughts and feelings as freely as possible.

### Quantitative measures: self-report questionnaire + time points

Selected psychological outcomes were collected at the beginning (Baseline – T0) and termination of the IRT intervention through self-reported measures (see Table 1) and at 1-month (T1) and 3-month follow-up (T2). Follow-up data were collected through an online survey. After T2, the psychologist who managed the intervention and Grace met in an online meeting to get feedback on her emotional, psychological, and sleep-related state.

**Table 1:** Measures.

Construct	Scale	Characteristics
Psychological changes in the aftermath of a trauma	Post-Traumatic Growth Inventory (PTGI; Prati and Pietrantoni, 2014)	It contains 21 items divided into five dimensions: relating to others (PTGI-RO), new possibilities (PTGI-NP), personal strength (PTGI-PS), spiritual change (PTGI-SC), and appreciation of life (PTGI-AL). Each item is scored on a scale of 0 (“never”) to 5 (“great degree”). The total score of the PTGI is the sum of all item scores. A higher score indicates trauma-related positive psychological changes.
Presence and intensity of daytime sleepiness	Epworth Sleepiness Scale (ESS; Johns, 1991)	It is an 8-item self-reported questionnaire. Each item is rated on a scale from 0 (“I have never dozed off”) to 3 (“high probability of dozing off”). The sum of the individual scores gives the overall score, which can range from 0 to 24. In general, ESS scores can be interpreted as follows: 0–5: Lower Normal Daytime Sleepiness; 6–10: Higher Normal Daytime Sleepiness; 11–12: Mild Excessive Daytime Sleepiness; 13–15: Moderate Excessive Daytime Sleepiness; 16–24: Severe Excessive Daytime Sleepiness.
Dream activity	Mannheim Dream Questionnaire (MADRE; Settineri et al., 2019)	It is a 20-item self-reported questionnaire that measures (a) dream-recall frequency (item 1) rated by a 7-point scale (0 = never and 6 = almost every morning); (b) emotional intensity of dream contents (item 2) rated by a 5-point scale (0 = not at all intense and 4 = very intense); (c) emotional tone (item 3) rated by a 5-point scale (−2 = very negative and 2 = very positive); (d) nightmare frequency (item 4) rated by an 8-point scale (0 = never and 8 = several times a week); (e) nightmare distress (item 5) rated by a 5-point scale (0 = not at all distressing and 4 = very distressing); and (f) lucid-dream frequency (item 10) rated by an 8-point scale (0 = never and 8 = several times a week). The frequency is asked concerning the previous month. Further, the scale assesses individual’ attitudes towards dreams by item 12 consisting of eight sentences with a 5-point format (0 = not at all and 4 = totally) and the impact of dream on daily life (the frequency of dream-sharing, the recording of dreams, the dreams affecting daytime mood, the creative dreams, and the problem-solving dreams) is assessed with items 13 to 17. Dream variables eliciting utilization of dreams (i.e., frequency of dream-sharing, recording of dreams, dreams affecting daytime mood, creative dreams, problem-solving dreams) are in an 8-point scale with 0 = never and 8 = several times a week. The questionnaire allowed us to also collect information on recurring nightmares (items 6 and 7), deja-vu experiences based on dreams (item 18), age of first lucid dream (item 11), reading about dreams (item 19, rated on a 3-point scale), and helpful dream literature (item 20, rated in a 5-point scale). For this study items 3, 4, and 5 are used.
Sleep disturbances	Pittsburgh Sleep Quality Index (PSQI; Buysse et al., 1989; Italian version by Curcio et al., 2013)	It is a 19-items self-reported questionnaire used to assess sleep quality during the last month. A global score greater than 5 is indicative of relevant sleep disturbances.
Aspects of sleep and dreams	Diary of sleep and dreams (adapted from Carney et al., 2012; Scarpelli et al., 2022c)	It is a self-report questionnaire compiled in 7 days in which the subject indicates aspects of sleep (presence and frequency of naps during the day, daily intake of medication, level of tiredness from 0 to 4 (0 = not at all; 4 = very much), time of going to bed, time taken to fall asleep, any nocturnal waking up, total hours of sleep, how rested they feel when they wake up, satisfaction with sleep and any disturbances during sleep) and aspects of dreams (if the subject has dreamt, and remembers the content, describe the content, presence of lucid dreams). For this study, only the sleep efficiency has been calculated.
Symptoms of common mental health status	The Depression Anxiety Stress Scale–21 (DASS-21; Lovibond and Lovibond, 1995)	It is a self-report measure in which the frequency and severity of depression, anxiety, and stress (emotional reactions) are assessed. Depression refers to dysphoria, anhedonia, lack of incentive, and low self-esteem; anxiety refers to somatic and subjective symptoms of anxiety and an acute response of fear; and stress evaluate irritability, impatience, tension, and persistent arousal. Subscale scores are calculated as the sum of the responses to the seven items from each subscale multiplied by 2 to suit the original 42 items. The cutoffs for severe depression, anxiety, and stress are ≥21, ≥15, and ≥ 26, respectively (Lovibond and Lovibond, 1995).

## Therapeutic intervention

**Figure 1:**
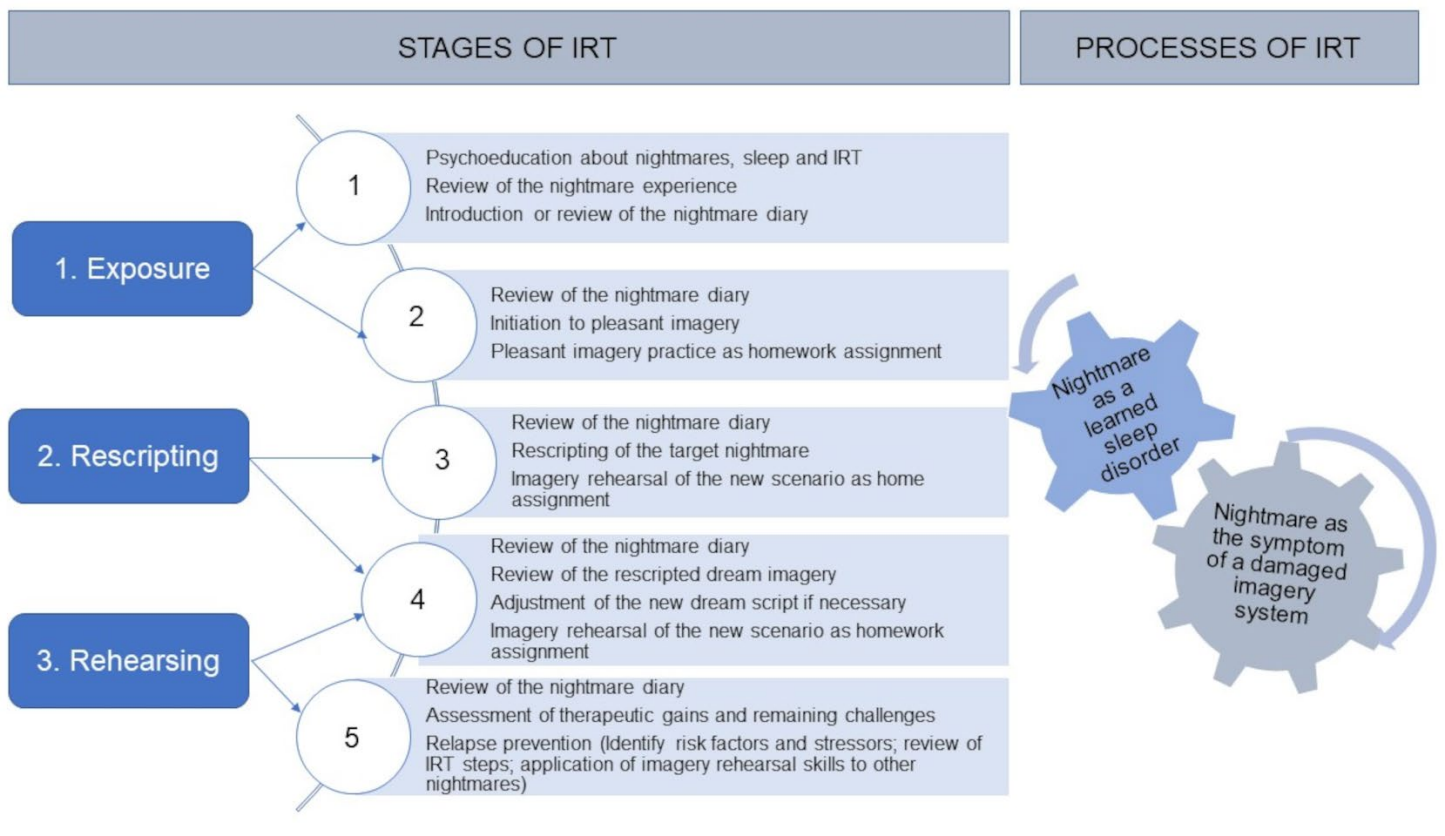
Stages and process of IRT. Imagery Rehearsal Therapy (IRT) is a brief evidence-based cognitive behavior therapy (CBT) technique developed by Kellner et al. (1992), which is effective in reducing nightmare distress and nightmares frequency, including PTSD-related forms of nightmares, and maintaining changes in long-term follow-up (Kunze et al., 2016; Ellis et al., 2019; Lancee et al., 2021). It works by inhibiting the original nightmare and providing a cognitive shift that empirically refutes the original premise of the nightmare (Lancee et al., 2011; Germain, 2013). Many non-pharmacologic techniques have been proposed to treat PTSD-related or idiopathic nightmares, including hypnosis, lucid dreaming, eye movement desensitization and reprocessing, desensitization, and IRT. However, only desensitization and IRT have been the object of controlled studies, and IRT has received the most empirical support (Krakow and Zadra, 2010). This method seems to have a beneficial effect on PTSD symptoms, the quality of sleep, and the nightmare frequency (Van Schagen et al., 2015; Van Schagen and Lancee, 2018).

Figure 1 shows the identified stages and process of IRT. Table 2 describes the IRT intervention.

**Table 2:** Description of IRT intervention.

First session: building therapeutic alliance and developing treatment credibility	The psychologist during the first session focused on building a positive relationship with the patient while normalizing arising trepidation and anxiety. Next, to help Grace “to emotionally elaborate herself from that life-changing event [i.e., the admission to ICU],” the psychologist asked Grace to write down the narrative or the central elements of the nightmare, describing in detail what happened in the dream and to whom. This technique of expressive writing was used to capture the most frightening elements of the dream on paper: the actual injury or death, horrific images or sounds, and the events leading up to the dramatic ending. “Doctors arrive at my bedside holding a tablet. Already from there, I can tell that something is wrong. They say, “Happy New Year,” and then they say that I have gotten worse and that unfortunately, they have to intubate me. There are three doctors and there are also two other ladies in the room with oxygen. The room is ugly, I am on a giant bed with wires and machinery, it was dark and scary. To my right was a clear glass window. I could see the doctor talking to the other doctors and looking at me worriedly. They hand me the tablet and tell me to call my family members to say hello before being intubated. I do not want to and start screaming and crying. My husband answers, wishing me a happy new year, but I burst into tears. There he realizes the situation has precipitated and cannot tell me anything, he shows me my children who start crying. Then I wake up.” Then, the psychologist discussed with Grace the link between nightmares and insomnia with two purposes: first, to make her feel that the focus of the intervention was on her sleep problems rather than on trauma or PTSD; second, to create a “mini-aha” experience (i.e., an insight, a sudden, conscious change in a person’s representation of a stimulus, situation, event, or problem) because most people who experienced trauma do not generally connect their nightmares to insomnia. This discussion also led to further considerations about sleep-disordered breathing, which is found in an alarming rate of trauma survivors with nightmares and PTSD (Krakow et al., 2000, 2001a,b; Krakow and Zadra, 2010). The discussion aimed to normalize her situation centers on the following key points: (a) nightmares fragment sleep; (b) sleep fragmentation causes poor sleep quality; (c) poor sleep quality is both a psychological and physiological process; (d) efforts to improve sleep quality provide the greatest relief from sleep problems; and (e) treating nightmares is an important step, and sometimes the best first step, in the treatment of posttraumatic sleep disturbance. Grace reported avoiding sleep onset at bedtime and re-onset in the middle of the night to prevent future nightmares. The psychologist discussed sleep avoidance as a conscious process with the patient. In addition, she described the progression of nightmares from an acute phase to a chronic disorder, as well as their adaptive function in motivating individuals to alter their behaviors or other aspects of their lives to avoid danger (Barrett, 1996). In accordance with this hypothesis, Grace was asked, “In your opinion, what is the function of the nightmare?” Grace began to consider that nightmares might have some benefit, but she still wasn’t able to acknowledge any. The first session ended by considering that nightmares can take on their own life.
Second session: nightmare as a learned and adaptive behavior	“What is the best explanation for the persistence of nightmares and disturbing dreams following stressful experiences or traumatic exposure?” the psychologist asked Grace. She answered that nightmares are a long-term consequence of trauma. Then, the professional and the patient proceeded to discuss the positive role of nightmares in releasing negative emotions resulting from the trauma during the night. By doing so, these emotions do not emerge during the day, allowing her to carry out daily activities. Grace was amazed by this new understanding, and a “mini-aha” experience re-occurred as she felt differently toward the nightmare as something beneficial. This corrective emotional experience also made her feel stronger, as the bad dream was no longer something she suffered from but a strategy she functionally implemented to defend herself (Alexander, 1993). Then, the discussion with Grace moved to principles of general imagery and pleasant imagery aimed at stimulating imaginative capacity through positive images as part of the IRT model: (a) imagery is a natural part of mental activity, which is easily described in behavioral terms as one component of the mental system of thoughts, feelings, and images; (b) imagery is often the last conscious activity before sleeping; (c) imagery during the day may be a bridge to imagery at night (dreams); (d) imagery is not meditation but simply a daydream with a bit more intention or structure as needed or desired; (e) imagery skills can be tested in brief exercises of a few minutes, and most people who experienced trauma have a reasonable ability to conduct such tests in groups or individually; (f) some people who experienced trauma are surprised of their healthy capacity to image things; and (g) most patients with non-extreme severe PTSD can easily practice pleasant imagery exercises at home. Trying to initiate a positive imagination process in a protected context such as the therapeutic setting, the psychologist asked Grace to think about something she would like to do for a long time until the next session. Grace stated that she was not lacking in imaginative abilities and – smiling – said *“to have her head among the clouds.”* At the end of the session, the patient was instructed to practice pleasant imagery every day for a few minutes until the next session.
Third session: imagery in the process of change	The third session began by discussing with Grace that many people who experience disturbing dreams might develop an imbalance in their thoughts, feelings, and imagery system: they might “think” too much and be detached from their unpleasant feelings and images. In addition, the expert described the significance and utility of imagery in everyday life, particularly in the process of change, and introduced the concept of rehearsal. Humans constantly engage in imagery rehearsal as they “practice” anticipated behaviors or experiences by imagining themselves in various new or familiar situations to determine how they might behave. Grace was asked to select an aspect of her life that she would like to change, with the only indication to choose something positive or neutral to change that would not elicit unpleasant feelings. Grace was amused by the exercise, which she carried out very calmly. At the end of the exercise, it was reasoned that dreams often draw their content from what happened during the day (daytime rest). Grace was astonished by this new learning, and she acknowledged that during the day she tended to avoid talking about COVID-19. Hearing people talk about the pandemic still afflicting the country also generated psychological tension that she physically needed to move away from them. “*They do not know what it was like for me*” added Grace crying, and concluded associating her avoidance behaviors to the traumatic experience. Then, she was informed that her daily avoidance behaviors combined with her limited imaginative activity led to traumatic nightmares. Grace now understood the function and operation of her nightmare and realized she required assistance. It was recommended to continue engaging in the imagination exercise for 5 to 10 min per day throughout the week and to reflect daily on the possibility of becoming a dreamer with the ability to have pleasant dreams rather than nightmares.
Session four: rejoice in your re-dreaming	In the fourth and last session, Neidhardt’s variation of “change the nightmare any way you wish” was employed (Krakow and Neidhardt, 1992; Neidhardt et al., 1992). Grace was instructed to rewrite the dream with a positive ending. This required some imagination, but it was specified that it could be accomplished by recalling heroic tales of survival from literature, film, or the media. The story could be outlandish, introduce rescuers, invoke her Super Hero superpowers, or realistically use self-defense, martial arts, weaponry, and/or the help of well-trained defenders such as the military or law enforcement combining autobiographical and fictional narrative (Barbieri and Musetti, 2018). Grace wrote the following: *“The doctors arrive and tell me I have nothing and let me go home. The room comes to life and flowers sprout from the floor. I am overjoyed and cannot wait to see my loved ones. Once back home, I hug my children tightly and make a delicious cake with them to forget the day at the hospital. I start living my life again but with a different perspective, treasuring that experience.”* Then, using the IRT protocol, the concept of changing the nightmare in any way desired was introduced. The steps below have elements of a technique borrowed from a phenomenon called lucid dreaming. Grace was asked before falling asleep to induce the intention to re-dream. She was instructed to repeat the procedure every time you have a nightmare or fear a recurrence. Grace reported at the conclusion of the session that she was relieved to have experienced no additional nightmares during the previous week.

## Follow-up and outcomes

Table 3 shows the results of the IRT-based psychological intervention between the three waves and the changes in the patient with respect to psychological and sleep-related variables.

**Table 3:** Clinical findings across time points.

	T0	T1	T2
PTGI (total score)	26	82	78
PTGI relating to others	21	47	40
PTGI new possibilities	0	20	20
PTGI appreciation of life	8	15	15
PTGI spiritual change	0	0	5
ESS	7	12	8
PSQI	12	8	8
MADRE			
Emotional intensity	4	2	2
Nightmare frequency	6	4	4
Sleep efficiency (from the Sleep and dreams diary)	84.86%	78.79%	
DASS stress	0	4	9
DASS anxiety	6	2	11
DASS depression	0	5	4

Furthermore, intervention sessions faithfully followed the IRT reference manuals (Kellner et al., 1992), and regular supervision sessions were conducted between the psychologist and experts in IRT (CF and SS) to ensure treatment fidelity.

### Post-traumatic growth

The total score of PTG increased from the baseline to follow-ups. According to Mazor et al. (2016), scores of 45 and below represented none to low PTG levels, whereas scores of 46 and above-represented medium to very high PTG levels. Before the treatment, Grace reported low levels of PTG; at the end of the treatment, and 4 months later, she showed high levels of PTG referring to the cutoff point of the scale. Furthermore, analyzing the five dimensions of PTGI, the patient showed an increase in value from the baseline to follow-ups in relating to others, new possibilities, appreciation of life, and spiritual change. Regarding the dimension of personal strength, Grace showed an increase in the long term, but the change was smaller than in previous subscales. Because there are no studies to our knowledge with respect to subdimension cutoffs, we cannot make assumptions regarding the minimal clinical difference.

### Daytime sleepiness

Regarding the daytime sleepiness measured with the Epworth Sleepiness Scale (ESS), the patient showed a higher normal daytime sleepiness in the baseline according to the scale range of the ESS; the score was higher at the end of the treatment, showing mild excessive daytime sleepiness and the daytime sleepiness at T2 returned higher normal also. Hence, daytime sleepiness showed no trend over the course of the study. It should be reiterated that the patient was being treated for OSAS and was following CPAP therapy at the time of treatment.

### Sleep quality

Sleep quality was measured using the Pittsburgh Sleep Quality Index (PSQI). In terms of self-reported sleep quality (i.e., scores at PSQI), the total PSQI score at the baseline was 12, with a cutoff of ≥5 indicating sleep disorders. Interestingly, the total PSQI decreased from the baseline in both follow-ups, meeting the minimal clinically important difference of ≥3 as suggested by previous evidence (Hughes et al., 2009; Eadie et al., 2013; McDonnell et al., 2014; Weinberg et al., 2020). In fact, a change of three points or more was chosen to indicate the smallest amount of change in PSQI that might be considered important by the patient according to the literature also considering the short-term assessment.

### Aspects of sleep and dreams

Sleep efficiency was measured with the sleep and dreams diary at T0 and T1, and it had a range from 93 to 63%, and there was no clear trend of improvement. Normal sleep efficiency is considered to be 85% or greater, and the patient thus appeared to show a wide range of variability (Miller et al., 2014), suggesting no normal sleep efficiency.

### Dream activity

Scores on the item related to the emotional intensity of dreams decreased at T1 and T2 (2 = rather intense dreams) compared to T0 (4 = very intense), while the emotional intensity remained unchanged with a score of 0 indicating a neutral tone. Moreover, the nightmare frequency at T0 was 6 (indicating a nightmare once a week), and at T1 and T2 was 4 (indicating a nightmare once a month). Despite the reduction in frequency, the emotional distress associated with the nightmare remained stable (i.e., quite distressing).

### Symptoms of common mental health status

Grace did not show improvement in stress, anxiety, and depression measured with DASS. In particular, the stress level showed a rising trend from the baseline to follow-ups and, as compared to the clinical cutoff point, the levels of stress were low in all three assessments; the anxiety showed a non-linear variation, and it was higher in T2 after 4 months from the beginning of treatment but always behind the clinical cutoff point. Moreover, for depression, there was an increasing trend from the baseline to follow-ups, but the levels were behind the clinical cutoff points in all the measurements.

## Discussion

Cognitive-behavioral treatments such as IRT have successfully reduced nightmare frequency, PTSD severity, and other mental health issues, such as depressive symptoms, while improving sleep quality (Ellis et al., 2019; Gieselmann et al., 2019). To date, to the best of our knowledge, no studies have investigated the efficacy of IRT either in the treatment of trauma-related experiences of COVID-19 or in patients with COVID-19 admitted to ICU. For these reasons, an IRT-based intervention protocol was conceptualized and administered to a patient with COVID-19-related nightmares and symptoms of PTSD linked to the experience of being admitted to the ICU 9 months after the traumatic event.

A preliminary semi-structured interview revealed anxiety and guilt over the possibility of having infected someone to be her first emotional reaction to the COVID-19 diagnosis. Furthermore, the need to be intubated and the risk of death shocked Grace to the point that it was difficult for the patient to describe her emotions. However, she was not the only one affected by this event as also her family struggled with this news and had to establish a new routine after the patient was discharged from the hospital. Nonetheless, the patient was able to find the silver lining in this traumatic experience and reported that exposure to a life-threatening illness reawakened her will to live. In fact, in addition to the difficulties people face as a result of traumatic events, a growing body of research indicates they might also experience positive life changes (Schaefer and Moos, 1992; Tedeschi and Calhoun, 1995). These experiences can be associated with the discovery of new coping strategies and personal and environmental resources in response to adverse life events (Prati and Pietrantoni, 2006)—a phenomenon named post-traumatic growth (PTG) (e.g., Lee et al., 2010; Wlodarczyk et al., 2016; Gil-González et al., 2022). PTG is linked to lower levels of depression (Helgeson et al., 2006), higher satisfaction with life (Mols et al., 2009), optimism, and positive wellbeing (Helgeson et al., 2006).

What emerged in the interview with Grace’s words was in line also with levels of PTG total score, and subscales (relating to others, new possibilities, appreciation of life, and spiritual change) increased after the IRT treatment in both follow-ups; in particular for the total score, the increase between T0 and T2 is more than 3-fold higher than the increase from T1 to T2.

Surprisingly, despite the IRT being an effective and specific treatment for trauma-related sleep disturbance and post-traumatic stress (see Casement and Swanson, 2012 for a meta-analysis), to the best of our knowledge, no studies have investigated the effect of this treatment on PTG. Implications for future research design and interpretation of published research will be implemented.

Regarding Grace’s daytime sleepiness during and after the treatment, results may suggest that it was high-normal across the time points. There was not a trend that could suggest a change, maybe because the patient followed therapy with CPAP for OSAS. Despite the literature showing several psychological barriers to CPAP treatment (Rapelli et al., 2021, 2022), Grace reported a good adjustment to it.

Consistent with previous findings, sleep quality improved following IRT treatment (Casement and Swanson, 2012; Albanese et al., 2022). Interestingly, the total PSQI score decreased from 12 (T0) to 8 (T1 and T2), meeting the minimal clinically significant difference of 3, as suggested by prior research (Longo et al., 2021). This improvement in sleep quality was accompanied by a decrease in the emotional intensity and frequency of nightmares measured with the MADRE. However, the level of nightmares-associated emotional distress did not significantly change across time points. The patient reported a neutral emotional state and a moderate level of distress related to nightmares (score 3 = quite distressing) across time points. These findings are different from the literature (Krakow et al., 2001b; Hansen et al., 2013); however, since this article presents a single case study, there is variability due to the involvement of a single subject which does not allow us generalizability. A possible explanation for this finding might be the increase in anxiety, depression, and stress symptoms measured with DASS at the 3-month follow-up and reported by Grace also in the final debriefing: the daily stress with the return to home and work could have had a negative impact on the psychological status. In fact, following her hospitalization and subsequent return home, the patient did not return to the clinic. However, due to divorce, we hypothesize that her psychological symptoms are more related to this stressful situation in the family than to her sleep concerns and post-traumatic events. In the literature, there were contrasting results regarding the effect of IRT on psychological well-being. For example, Lu et al. (2009) did not find improvement in depression after the IRT for war veterans; in contrast, Simard and Nielsen (2009) found that administering a single session of IRT to a group of children with sleep disturbances, but without PTSD diagnosis, led to a reduction in the distress caused by nightmares and to a decrease of other anxious and depressive symptoms.

A limitation of this case study might be that the duration of the intervention was limited to four sessions; in fact, a larger number of sessions would have potentially promoted further improvement in the patient’s outcome; however, this case reflects the common, real-world practice and is also illustrative of successful work with complex patients who complain of significant psychological and physical difficulties. Furthermore, since this article presents a single case study, there is variability due to the involvement of a single subject which does not allow us generalizability. Moreover, since there are no controls, we could not assume that the IRT intervention is more effective than usual care.

This case study suggests that the use of IRT may help to reduce the frequency of nightmares and improve sleep quality in a female patient after ICU for COVID-19. Although additional research is warranted on the specific impact of IRT treatment on PTSD and sleep disorders among patients who experience COVID-19 in an ICU, our case suggests that IRT may be an effective treatment for adults experiencing sleep disturbances and post-traumatic symptoms after admission to an ICU for COVID-19.

## Patient perspective

Grace at the end of the intervention trusted the method, reporting that she was happy not to have experienced further nightmares related to the ICU. She said she is more aware of her own way of experiencing the emotions related to that traumatic event and reiterated that she is surprised at herself that she was able to give positive growth meaning to the event.

## Data availability statement

The raw data supporting the conclusions of this article will be made available by the authors, without undue reservation.

## Ethics statement

The studies involving human participants were reviewed and approved by Medical Ethics Committee of the IRCCS Istituto Auxologico Italiano (ID: 2021_03_23_02). The patients/participants provided their written informed consent to participate in this study. Written informed consent was obtained from the individual(s) for the publication of any potentially identifiable images or data included in this article.

## Author contributions

GR, GV, SS, GPi, AM, GPl, CF, and GC contributed to the development of the study. GR, GV, GPi, and CF contributed to the analysis of the results. All authors contributed to the article and approved the submitted version.

## Funding

The research study was funded by the Italian Ministry of Health.

## Conflict of interest

The authors declare that the research was conducted in the absence of any commercial or financial relationships that could be construed as a potential conflict of interest.

The reviewer EV declared a past co-authorship with the authors AM, GPl, CF, and GC to the handling editor.

## Publisher’s note

All claims expressed in this article are solely those of the authors and do not necessarily represent those of their affiliated organizations, or those of the publisher, the editors and the reviewers. Any product that may be evaluated in this article, or claim that may be made by its manufacturer, is not guaranteed or endorsed by the publisher.

## Supplementary material

The Supplementary material for this article can be found online at: https://www.frontiersin.org/articles/10.3389/fpsyg.2023.1144087/full#supplementary-material
