# Supplementary material for: The long wave of COVID-19: a case report using Imagery Rehearsal Therapy for COVID-19-related nightmares after admission to intensive care unit
Source: Front Psychol. 2023 May 18;14:1144087. doi: 10.3389/fpsyg.2023.1144087 (PMC10232986; doi:10.3389/fpsyg.2023.1144087)
Supplement: Supplementary file 1 [file Data_Sheet_1.PDF]

CARE Checklist of information to include when writing a case report

CARE case report guidelines

(cc) BY-NC-ND

Topic	Item	Checklist item description	Reported on Line
Title	1	The diagnosis or intervention of primary focus followed by the words "case report"	1-2
Key Words	2	2 to 5 key words that identify diagnoses or interventions in this case report, including "case report"	27-28
Abstract (no references)	3a	Introduction: What is unique about this case and what does it add to the scientific literature?	6-11
	3b	Main symptoms and/or important clinical findings	12-17
	3c	The main diagnoses, therapeutic interventions, and outcomes	17-20
	3d	Conclusion—What is the main "take-away" lesson(s) from this case?	21-26
Introduction	4	One or two paragraphs summarizing why this case is unique (**may include references**)	62-74
Patient Information	5a	De-identified patient specific information.	77
	5b	Primary concerns and symptoms of the patient.	77-87
	5c	Medical, family, and psycho-social history including relevant genetic information	77-87
	5d	Relevant past interventions with outcomes	87
Clinical Findings	6	Describe significant physical examination (PE) and important clinical findings.	77-87
Timeline	7	Historical and current information from this episode of care organized as a timeline	77-87
Diagnostic Assessment	8a	Diagnostic testing (such as PE, laboratory testing, imaging, surveys).	94-125
	8b	Diagnostic challenges (such as access to testing, financial, or cultural)	94-125
	8c	Diagnosis (including other diagnoses considered)	94-125
	8d	Prognosis (such as staging in oncology) where applicable	94-125
Therapeutic Intervention	9a	Types of therapeutic intervention (such as pharmacologic, surgical, preventive, self-care)	121-125 + Figure 1 and Table 2
	9b	Administration of therapeutic intervention (such as dosage, strength, duration)	121-125 + Figure 1 and Table 2
	9c	Changes in therapeutic intervention (with rationale)	121-125 + Figure 1 and Table 2
Follow-up and Outcomes	10a	Clinician and patient-assessed outcomes (if available)	127-186 + Table 3
	10b	Important follow-up diagnostic and other test results	127-186 + Table 3
	10c	Intervention adherence and tolerability (How was this assessed?)	131-133
	10d	Adverse and unanticipated events	134-136
Discussion	11a	A scientific discussion of the strengths AND limitations associated with this case report	248-251
	11b	Discussion of the relevant medical literature **with references**.	188-247
	11c	The scientific rationale for any conclusions (including assessment of possible causes)	252-256
	11d	The primary "take-away" lessons of this case report (without references) in a one paragraph conclusion	252-256
Patient Perspective	12	The patient should share their perspective in one to two paragraphs on the treatment(s) they received	260-264
Informed Consent	13	Did the patient give informed consent? Please provide if requested	Yes ☒ No ☐
