# Supplementary material for: The long wave of COVID-19: a case report using Imagery Rehearsal Therapy for COVID-19-related nightmares after admission to intensive care unit
Source: Front Psychol. 2023 May 18;14:1144087. doi: 10.3389/fpsyg.2023.1144087 (PMC10232986; doi:10.3389/fpsyg.2023.1144087)
Supplement: Supplementary file 2 [file Data_Sheet_2.docx]

**Qualitative analysis: The lived experience of being in ICU for COVID-19**

In this section, the results of the interview conducted with the patient in the first meeting with the psychologist at the beginning of the psychological intervention during admission to the rehabilitation program will be presented. The semi-structured interview occurred in a dedicated room of the hospital, lasted about 1 hour, and asked about the experience of being infected and the experience of the ICU, feelings, and worries about COVID-19 management during the hospitalization and at home, feelings about COVID-19 consequences during the hospitalization and at home after discharge, frequent nightmares related to the experience in ICU (Table 1).

The interview transcription was qualitatively analyzed using a phenomenological hermeneutic research design (Lindseth and Norberg, 2004) without using any software. A data-driven approach (versus theory-driven), considered the most effective way to investigate subjective experiences (Davidson et al., 2008), was used for text analysis. Since the analytic approach was inductive, the comparison with the research literature was conducted only at the end of the whole process as a sort of “return to the theory”. Two authors (GV and SS) independently performed the data analysis. Disagreements between coders were reconciled through extensive in-person discussion and with the whole multidisciplinary team, which enhanced the researchers’ reflexivity and thus reduced the influence of their preconceptions and biases on the analytic process, with increased rigor. The analytic process initially entailed line-by-line reading of the interview to provide a preliminary description of relevant topics, with notes recorded directly in the text. The interview, in a primary recursive phase, has been read and re-read with constant attention to the connection between themes and the whole corpus of the interview.

The congruence for each major theme was examined with independent evaluations by each coder followed by discussion and consensus during team meetings.

Table 2 summarized the main themes of the interview and significant quotations.

***Being positive for COVID-19: The world fell on me***

Finding out that you have been infected with a potentially deadly virus exposes you to the finite nature of life, especially at the start of the pandemic when mortality rates were very high in all age groups, and being positive for COVID-19 was a harbinger of death. Not only was it riskier to fall ill, and the symptoms more serious, but the hospitals were full and overcrowded, and the need to be admitted to the hospital was experienced with great anxiety. Patients may then have feelings of guilt about the possibility of having infected someone, especially fragile people.

When Grace found out that she had COVID-19, she was shocked and a sense of fear and helplessness overcame her. She also immediately showed concern about infecting her loved ones.

***Being intubated takes away your words***

When the situation is so serious that one needs to be intubated, the person realizes the potential risk of not making it. People may feel that they have reached the end of their life as if there is no escape and that being intubated is the anteroom of death.

Grace had difficulty describing how she felt when she was told she needed to be intubated. Even after months, grace remembered that moment with intense fear while crying

***A warrior with a mask***

The COVID-19 experience can be taken as a non-normal and unexpected and potentially traumatic life event due to the characteristics of the worldwide catastrophic event and related symptoms. The potentially life-threatening experience of having had a covid and being intubated leaves a mark, but it also represents a watershed with one's former life, because it becomes an experience that marks and renews gratitude towards life. Having feared the worst and having risked dying contributes to having a new outlook on life and to making new plans.

Sometimes, however, it happens that you make yourself appear strong in the eyes of others, but inside you feel bad because the emotional impact of the event is very strong. Others may consider the person who won COVID-19 as a survivor, but a great psychological malaise may prevail instead.

Grace defined herself as a "warrior with a mask". A warrior because she won the battle with COVID-19, with the mask because she forced herself to appear strong to others despite her inner suffering.

***Long-covid syndrome: COVID-19 leaves its mark***

Having COVID-19 can cause disabling symptoms in some cases even months later. These symptoms have been defined as a long-COVID syndrome and in addition to symptoms of dyspnoea can include difficulties with memory and concentration, fatigue, insomnia, gastrointestinal problems, fever, menstrual cycle changes, and joint and muscle pain.

The severity of long-covid symptoms may be greater in those who have been in intensive care, and this is compounded by the difficulty of coping psychologically with the stressful and potentially traumatic situation of being intubated in intensive care. In fact, in some cases, the psychological consequences may relate to those of PTSS. It is not uncommon for patients with COVID-19 who have been in the ICU to experience nightmares, flashbacks, and avoidance.

Signs and symptoms of the traumatic experience were still alive in Grace's words and behavior. She experienced daily nightmares reliving the last minutes before being intubated, when she called the family to give her the news. She avoided talking about this topic with friends and family members because this causes her hyperarousal (sweating, palpitation and agitation).

***The family at home: reactions to communication***

The need for emergency intubation for COVID-19 also affects family members who remain at home and learn the news through a phone call. Sometimes the news is communicated to family members, including children, directly by the patient without mediation by doctors. Such communication can be enormously stressful because it exposes the communicating person to the difficulty of communicating bad news and of managing the emotional wave that such news brings, especially in the presence of children. On the part of family members, learning such news exposes them to the difficulty of not finding the right words and of feeling out of control of the situation, because they are far from their loved one and unprepared for what will happen.

Grace talked about the reaction of her family and said she was surprised for the emotional breakdown of the husband. The news of the intensive care significantly affected her husband and children.

***My unfamiliar family***

Returning home after a month in intensive care with your life hanging in the balance can be difficult. You have to rebuild your family's rhythms and come to terms with what has happened at your own pace. The children may also need psychological support to process the traumatic event that has entered their lives. The person who has returned home is no longer the same as the person he or she was before because this event has changed him or her.

Grace returned home but the month spent fighting between life and death changed her forever, and returning to her previous life was not as easy as she thought. Even the family members, who were looking forward to seeing her, had to face this change. The event is even more difficult for children to process.

| Table 1. Questions of the interview |
| --- |
| **Diagnosis - admission phase**  Can you tell me about your experience with COVID-19?  What were the main emotions you had when you were told you were infected?  What symptoms did you experience?  How did you react? How did you cope with the communication and then the admission?  Tell me about your feelings and psychological state when you were hospitalized. What psychological symptoms and perceptions do you have of the time you were hospitalized? |
| **Long – covid symptoms and consequences**  How have your habits changed as a result of COVID-19?  What has changed in your life?  How did you react to these changes in your life and work?  How has your sleep changed as a result of the COVID-19?  How do you rate your quality of life after COVID-19?  How do you assess your future?  How were your relationships with your family and friends during this illness?  What effect did COVID-19have on the family? What attitudes and behaviours did the family have towards you?  How has COVID-19affected your family and their social relationships? |
| **Nightmare**  Tell me about your recurring dream  How disabling is it for you? How much do you think about it during the day? How does it affect your daily life?  What emotions does it provoke in you?  How do you feel during the day after having this dream? |

Table 3. Qualitative Analysis

| **Themes** | **Significant quotations** |
| --- | --- |
| Being positive for COVID-19: The world fell on me | *“And my whole world fell apart, I saw the bottom of it. Yes, because I was more afraid for the children and for my husband.”*  *“Because my husband was operated and had some problems with his lungs so I was more afraid for him than for me, honestly. I say "it's not like I'm going to attack him" but fortunately he took it in a mild form.”* |
| Being intubated takes away your words | *“There... it's an emotion that I don't think I would ever wish on anyone. There I saw my life end. Just when I said goodbye to my family, I said to myself "it's over"… I can't explain it, it's really bad. Honestly, I can't even speak because (crying)…”* |
| A warrior with a mask | *“The experience gave me the strength to... that is, it marked me a lot. But it gave me the strength to want to live. To live every day... to live every day but I must live well. I have to feel good.”*  *“I felt really bad about it. Yes, and I say, I did... I said... I showed you in front of everybody and they said "No Grace you're fine, you see you're a warrior here and there" but let's say that it was a mask to say that everybody shows me that I'm strong but then I collapse.”* |
| Long-covid syndrome: COVID-19 leaves its mark | *“I went to cheer myself up and I couldn't do it, I had no strength and nothing. Then after a couple of days of being exercised on the bed, they tried to put me on my feet but my legs couldn't hold me and they gave in. I cried again because I thought I would never walk again, but slowly and patiently I managed it. But even there it's another very bad feeling because you return as when you were a child, you don't know how to do anything anymore. Not even eat. You have to be fed.”*  *“I mean, I'm always tired, always tired and... but I do everything for the children, always them, not to let them see it, but how I do things, for example, I take them to school, and then I arrive home and I have to rest, I can't do it at all and instead before I didn't, before I worked... I hope to go back to work soon because I'll resume my life too.”*  *“In the evening when I go to sleep I think... that is like when you relax, you have a moment of relaxation of the body, you have a moment when you go to sleep, you have these flashes. Just flashes of this tablet. They come there but then I tell them every day, it's not like you say…”*  *“No, I'm sorry because I don't want to, I mean I don't want to be a burden... honestly at home, they don't even know. I don't want to put more problems on top of more problems. I don't feel like talking about it yet. Honestly, I've only talked to you about it.”*  *“Before, a lady came near me, who always tells me that she has been in intensive care, and there I feel panic and I have to go away because those moments come back to my mind.”* |
| The family at home: reactions to communication | *“So they said "when are you coming home?" and I said "Nothing, unfortunately, they have to intubate me right away because otherwise there is no chance that I will make it" and then they started crying... my husband worse than the children. He didn't expect it and he had a big blow, he collapsed in front of me... I didn't think so because of his character. Afterward, he told me "I saw my life passing in front of me... there is..." he told me "I could not imagine a life without you".* |
| My unfamiliar family | *“Coming back home after only one month I saw them already grown up... it seemed a moment and I say but where have I been all this time?”*  *“My daughter was very grumpy with me. Because there were moments when I would say "What have you got Liza with me, why are you doing this". And she didn't say anything. Then, one day, she showed all her anger and said 'you were dying and you didn't tell us anything, do you think it is right to leave us here alone? Then she opened up and changed completely.”* |
